# The Orthodontist in Pediatric Sleep-Disordered Breathing: A Systematic Review and Clinical Practice Framework

**DOI:** 10.7759/cureus.115795

**Published:** 2026-09-04

**Authors:** Amey G Patil, Joseph Maawad, Peter Yacoub, Shimon Goldstein, Aishwarya S Karnik, Anil Ardeshna

**Affiliations:** 1 Department of Restorative Dentistry, Center for Temporomandibular Disorders and Orofacial Pain, Department of Diagnostic Sciences, Rutgers School of Dental Medicine, Rutgers, The State University of New Jersey, Newark, USA; 2 Department of Orthodontics, Rutgers School of Dental Medicine, Rutgers, The State University of New Jersey, Newark, USA; 3 Department of Pediatric Dentistry, Rutgers School of Dental Medicine, Rutgers, The State University of New Jersey, Newark, USA; 4 General Dentistry, Private Practice, Lakewood, USA; 5 General Dentistry, Private Practice, Mumbai, IND

**Keywords:** adenotonsillectomy, clinical decision framework, mandibular advancement, obstructive sleep apnea, orofacial pain, orthodontics, pediatric dentistry, polysomnography, rapid maxillary expansion, sleep-disordered breathing

## Abstract

Pediatric sleep-disordered breathing (SDB), including obstructive sleep apnea (OSA), is common and clinically consequential, and its craniofacial associations bring affected children into frequent contact with orthodontists. Whether and how orthodontists should participate in care is contested, with marketing claims sometimes outpacing evidence. We conducted a systematic review of pediatric orthodontic interventions for SDB and synthesized current professional guidance into a clinical practice framework. We conducted a systematic PubMed/MEDLINE search from January 1, 2001, to May 20, 2026, using terms for pediatric SDB and orthodontic intervention. We applied English-language and pediatric age filters. The review was not prospectively registered, and no protocol was published. Controlled studies reporting objective respiratory outcomes formed the primary evidence base, while uncontrolled pre-post studies were considered supportive. We assessed risk of bias using RoB 2 (parallel and crossover trials) and the National Heart, Lung, and Blood Institute (NHLBI) Before-After Quality Assessment Tool for uncontrolled pre-post studies. We summarized evidence certainty using a GRADE-informed approach. We included 16 reports representing 15 unique studies: seven controlled reports and nine uncontrolled pre-post reports from eight cohorts. Controlled studies comparing mandibular advancement or functional appliances with untreated or sham controls reported reductions in the apnea-hypopnea index in the treated groups. Controlled evidence for maxillary expansion was weaker and less consistent: in the only included trial with an untreated concurrent control, the apnea-hypopnea index fell comparably in treated and untreated children with no significant between-group difference, and two trials found maxillary expansion no better than, or inferior to, adenotonsillectomy. Studies were small and clinically heterogeneous, and complete variance data were not uniformly available. Quantitative pooling was therefore inappropriate and was not performed; findings are reported as a structured narrative synthesis. Across outcomes, certainty of evidence was low to very low, limited by risk of bias, imprecision, indirectness, and confounding by spontaneous resolution. Selected orthodontic interventions may improve respiratory indices in appropriately diagnosed children. Still, the evidence remains weak and does not establish equivalence with established physician-directed therapies or support orthodontic treatment to prevent future SDB. Consistent with American Association of Orthodontists (AAO) and American Academy of Pediatric Dentistry (AAPD) recommendations and, where applicable, informed by American Academy of Dental Sleep Medicine (AADSM)/American Academy of Sleep Medicine (AASM) dental sleep medicine guidance and American Academy of Orofacial Pain (AAOP) perspectives on interdisciplinary management, the orthodontist's evidence-based role is to identify at-risk children, conduct appropriate risk screening, and facilitate referral within an interdisciplinary, physician-led pathway. Physicians establish the diagnosis and direct medical management; polysomnography remains the diagnostic reference standard; and craniofacial imaging should not be used as a standalone screening or diagnostic test for pediatric SDB. This systematic review presents a guideline-anchored clinical decision framework to operationalize this role.

## Introduction and background

Pediatric obstructive sleep apnea (OSA) lies within the broader continuum of sleep-disordered breathing (SDB), ranging from primary snoring and upper-airway resistance to frank obstructive apnea. It affects an estimated 1-5% of children and, when untreated, is associated with cardiovascular, metabolic, growth, and neurobehavioral morbidity [1,2]. Adenotonsillar hypertrophy is the predominant anatomical contributor, and adenotonsillectomy is generally considered first-line therapy when clinically indicated; however, residual disease is common, and obesity and craniofacial morphology may modify both risk and treatment response [1,2].

Because skeletal and dental development is closely related to upper-airway form, children with SDB may present with craniofacial and occlusal features of interest to orthodontists, including maxillary transverse constriction, mandibular retrognathia, increased lower facial height, and Class II malocclusion. The cadence of orthodontic care, with repeated visits across developmental periods, places orthodontists in a useful position to recognize risk indicators and initiate appropriate referral [3,4]. This has fueled interest in “airway-focused” orthodontics, but also concern about overreach: procedures appropriate for screening have sometimes been used as if they were diagnostic, and appliances have been marketed as cures or preventive interventions, claims that exceed the current evidence [3,4]. Although changes in upper-airway dimensions following orthodontic therapy have been evaluated in patients with OSA, establishing their direct translation to objective respiratory improvement remains critical [5].

Professional bodies have responded. The American Association of Orthodontists (AAO) 2019 White Paper framed the orthodontist’s role as screening and referral, reserving diagnosis for physicians [3]. The 2026 AAO White Paper update broadened the terminology from OSA to SDB. It stated that current evidence does not support claims that orthodontic intervention prevents the development of SDB, that orthodontic procedures, including extractions, increase its likelihood, or that craniofacial imaging such as cone-beam computed tomography (CBCT) or lateral cephalograms provides reliable screening or diagnostic value for SDB [4]. The American Academy of Pediatric Dentistry (AAPD) similarly encourages screening and medical referral and recommends orthodontic assessment before appliance therapy when appropriate [6]. Guidance from the American Academy of Dental Sleep Medicine (AADSM), developed in collaboration with the American Academy of Sleep Medicine (AASM), primarily addresses oral appliance therapy in adults [7]. At the same time, the American Academy of Orofacial Pain (AAOP) situates sleep bruxism and orofacial pain within the same interdisciplinary care space [8].

Against this backdrop, two questions matter for practice. First, what does the controlled pediatric evidence show about the effect of orthodontic interventions on objective respiratory outcomes in children with SDB? Second, how should orthodontists act in a way that is consistent with both the evidence and current professional guidance? This systematic review addresses both questions by synthesizing the available pediatric interventional evidence and integrating current AAO and AAPD recommendations, together with relevant contextual guidance from AADSM and AAOP, into a practical clinical decision framework. Because the eligible studies were clinically and methodologically heterogeneous, the evidence is presented as a structured narrative synthesis rather than a quantitative meta-analysis.

## Review

Methods

Protocol and Reporting Standards

This systematic review was conducted and reported in accordance with the Preferred Reporting Items for Systematic Reviews and Meta-Analyses (PRISMA) 2020 statement [9]. We performed a systematic PubMed/MEDLINE search, followed by predefined population, intervention, comparator, outcomes, and study design (PICOS)-based study selection, standardized data extraction, design-specific risk-of-bias assessment, and a Grading of Recommendations Assessment, Development and Evaluation (GRADE)-informed certainty appraisal. Because the eligible studies were clinically and methodologically heterogeneous and did not provide sufficiently compatible data for defensible quantitative pooling, we synthesized the findings narratively. The review was not prospectively registered, and no protocol was published or otherwise made publicly available.

Eligibility Criteria (PICOS)

We defined eligibility criteria using the PICOS framework, as summarized in Table 1. We included studies of children and adolescents younger than 18 years with SDB or OSA who underwent orthodontic interventions, including rapid or slow maxillary expansion, functional or mandibular advancement appliances, twin-block therapy, monobloc therapy, or oral jaw-positioning appliances. Eligible comparators included untreated controls, sham appliances, observation or watchful waiting, adenotonsillectomy, or pre-treatment baseline.

**Table 1:** Eligibility Criteria (PICOS) AHI, apnea-hypopnea index; CBCT, cone-beam computed tomography; PICOS, population, intervention, comparator, outcomes, and study design; PSG, polysomnography; SDB, sleep-disordered breathing; TMJ, temporomandibular joint.

Element	Included	Excluded
Population	Children/adolescents younger than 18 years with sleep-disordered breathing or obstructive sleep apnea	Adults; syndromic or secondary populations primarily managed surgically, including Pierre Robin sequence, Marfan syndrome, and TMJ ankylosis
Intervention	Orthodontic or dentofacial orthopedic interventions, including rapid or slow maxillary expansion, functional/mandibular advancement appliances, twin-block therapy, monobloc therapy, and oral jaw-positioning appliances	Isolated non-orthodontic surgery, distraction osteogenesis as the primary intervention, and non-orthodontic therapies as the sole intervention
Comparator	Untreated control, observation/watchful waiting, sham appliance, adenotonsillectomy, or pre-treatment baseline	Not applicable
Outcomes	Primary: objective respiratory outcomes, including AHI, respiratory disturbance index, apnea index, hypopnea index, or oxygen saturation measured by PSG or a comparable sleep study. Secondary outcomes such as quality of life, airway volume, nasal resistance, or stability were considered only when a respiratory sleep outcome was reported.	Studies reporting only cephalometric, CBCT, or airway-dimensional surrogate outcomes without respiratory sleep outcomes
Study design	Randomized controlled trials, controlled clinical trials, prospective or retrospective comparative studies, and uncontrolled pre-post interventional studies with usable respiratory outcome data	Case reports, non-eligible descriptive case series, narrative reviews, expert opinion articles, editorials, and protocols without outcome data

The primary outcomes were objective respiratory outcomes, including the apnea-hypopnea index, respiratory disturbance index, apnea index, hypopnea index, and oxygen saturation, measured by polysomnography or a comparable sleep study. We excluded studies reporting only cephalometric or airway-dimensional surrogate outcomes without respiratory sleep outcomes. We excluded adult-only studies, syndromic or surgically managed secondary populations, isolated non-orthodontic surgical interventions, protocols without results, case reports, narrative reviews, editorials, and expert-opinion articles.

Information Sources and Search

We conducted a systematic search in PubMed/MEDLINE for studies published from January 1, 2001, through May 20, 2026. We selected PubMed/MEDLINE as the sole bibliographic database because it provides broad coverage of biomedical, pediatric, sleep-medicine, dental, and orthodontic literature and supports a reproducible search using both MeSH terms and free-text terms. The review was designed as a focused synthesis of clinically relevant interventional evidence rather than an exhaustive multi-database search, and we acknowledge that relevant studies indexed exclusively in other databases may therefore have been missed.

The search combined three concept blocks: pediatric population, SDB or OSA, and orthodontic intervention. Search terms included MeSH terms and free-text terms related to children and adolescents, obstructive sleep apnea, sleep-disordered breathing, snoring, apnea-hypopnea index, palatal expansion, rapid maxillary expansion, mandibular advancement, functional appliances, twin-block therapy, oral appliances, and myofunctional therapy. Table 2 provides the full PubMed/MEDLINE search strategy.

**Table 2:** PubMed/MEDLINE Search Strategy MeSH, Medical Subject Headings; OSA, obstructive sleep apnea; SDB, sleep-disordered breathing.

Source	Strategy / status
PubMed/MEDLINE	A systematic PubMed/MEDLINE search was conducted using three concept blocks combined with AND: pediatric population, sleep-disordered breathing/obstructive sleep apnea, and orthodontic interventions. The search included MeSH terms and free-text terms for children/adolescents, obstructive sleep apnea, sleep-disordered breathing, snoring, apnea-hypopnea outcomes, palatal/maxillary expansion, mandibular advancement, functional appliances, oral appliances, twin-block therapy, Herbst appliance, growth modification, and myofunctional therapy.
Full search string	((“Child”[Mesh] OR “Adolescent”[Mesh] OR “Infant”[Mesh] OR child*[tiab] OR children[tiab] OR pediatric*[tiab] OR paediatric*[tiab] OR adolescen*[tiab] OR juvenile*[tiab] OR youth[tiab] OR “growing patient*”[tiab]) AND (“Sleep Apnea, Obstructive”[Mesh] OR “Sleep Apnea Syndromes”[Mesh] OR “Snoring”[Mesh] OR “Sleep-Disordered Breathing”[tiab] OR “sleep disordered breathing”[tiab] OR “sleep apnea”[tiab] OR “sleep apnoea”[tiab] OR OSA[tiab] OR OSAS[tiab] OR OSAHS[tiab] OR SDB[tiab] OR snoring[tiab] OR “apnea-hypopnea”[tiab] OR “apnoea-hypopnoea”[tiab] OR “respiratory disturbance index”[tiab]) AND (“Palatal Expansion Technique”[Mesh] OR “Orthodontic Appliances, Functional”[Mesh] OR “Orthodontics”[Mesh] OR “Mandibular Advancement”[Mesh] OR “Occlusal Splints”[Mesh] OR “rapid maxillary expansion”[tiab] OR “rapid palatal expansion”[tiab] OR “maxillary expansion”[tiab] OR “palatal expansion”[tiab] OR RME[tiab] OR RPE[tiab] OR “slow maxillary expansion”[tiab] OR “functional appliance*”[tiab] OR “mandibular advancement”[tiab] OR “oral appliance*”[tiab] OR orthodontic*[tiab] OR “twin block”[tiab] OR Herbst[tiab] OR “growth modification”[tiab] OR myofunctional[tiab]))
Limits	English language; age birth-18 years.
Search period	January 1, 2001, to May 20, 2026.
Results	Initial records: 728; after English-language limit: 674 (54 records removed); after pediatric age limit: 514 (a further 160 records removed).

We applied predefined English-language and pediatric-age limits within the database. We applied the English-language restriction because resources for reliable translation and verification of non-English full-text reports were not available. The search retrieved 728 records before application of these limits. The English-language limit removed 54 records, and the pediatric-age limit removed a further 160 records, leaving 514 records for screening. No registers, trial registries, websites, organizations, reference lists, or citation searches were used as additional sources of records during the primary selection process.

The January 1, 2001, start date was chosen to focus the review on contemporary pediatric SDB and orthodontic interventional literature most applicable to current clinical practice. The final database search was conducted on May 20, 2026.

Study Selection and Data Extraction

Records identified through PubMed/MEDLINE were screened against predefined eligibility criteria, and potentially relevant reports underwent full-text assessment. Three reviewers participated in study selection and data extraction using a standardized extraction framework. Screening and extraction were performed collaboratively rather than independently in duplicate. The reviewer group subsequently reviewed and verified eligibility decisions and extracted outcome data against the full-text reports. The reviewer group discussed and resolved any discrepancies, uncertainties, or differences in interpretation by consensus. Extracted variables included study design, population characteristics, intervention and comparator details, sample size, participant age, follow-up duration, respiratory outcome measures, and reported treatment effects. Controlled studies with a concurrent comparator constituted the primary evidence base. Uncontrolled pre-post studies were retained as supportive evidence because their findings are more vulnerable to confounding by growth, regression to the mean, concurrent care, and spontaneous improvement.

Studies were classified as controlled or uncontrolled based on whether a concurrent comparator group contributed data for the respiratory outcome, rather than on the design label used by the original authors. Two reports described a comparison group assessed only for craniofacial or airway-dimensional outcomes and did not undergo a repeat sleep study; these were classified as uncontrolled for this review.

Two publications by Villa et al., published in 2007 and 2015, were identified as reports from a partially overlapping cohort based on the explicit description and study-flow diagram in the 2015 publication. The 2007 report enrolled 16 participants, of whom 14 completed follow-up. The 2015 report continued the earlier protocol and added 26 newly completed participants to those 14 participants, yielding a combined cohort of 40. We therefore retained the publications as separate reports but treated them as one study for overall study counts and certainty assessments. We extracted outcomes or follow-up periods unique to each publication separately; however, we did not count overlapping participants twice, and we did not treat the two reports as independent datasets in the synthesis.

Risk of Bias and Certainty

We assessed risk of bias using design-specific instruments. Individually randomized parallel-group trials were assessed with the Cochrane risk-of-bias tool for randomized trials (RoB 2) [10]. The single randomized crossover trial was assessed with the RoB 2 extension for crossover trials, which retains the five standard RoB 2 domains and adds a domain addressing bias arising from period and carryover effects [11]. Uncontrolled before-after studies without a concurrent control group were appraised with the National Heart, Lung, and Blood Institute (NHLBI) Quality Assessment Tool for Before-After (Pre-Post) Studies With No Control Group [12]. The NHLBI states that these tools have not been independently published and are therefore not considered standardized; as a result, the resulting ratings are reported as structured quality judgments rather than validated risk-of-bias estimates.

The Risk Of Bias In Non-randomized Studies - of Interventions (ROBINS-I) tool was not applied, because no included study met its scope: on full-text assessment, every included report with a concurrent comparator for the respiratory outcome was described by its authors as randomized, and every non-randomized report lacked a concurrent respiratory comparator and was therefore appraised as an uncontrolled before-after study.

Two trials randomized participants to a treatment sequence rather than to a single intervention. Because both delivered irreversible or persistent interventions with no possible washout, and because, in one, the second intervention was administered only to participants with a residual apnea-hypopnea index above one event per hour, neither could provide an uncontaminated within-person treatment comparison. Both were therefore assessed with RoB 2 as randomized parallel-group comparisons of treatment strategies, judged on outcomes after the first assigned intervention; their second-period and end-of-trial results are reported as supportive longitudinal evidence rather than as randomized comparative evidence.

Assessments were made at the level of the respiratory outcome relevant to this review, and disagreements were resolved through discussion and consensus. Certainty of evidence was summarized by outcome using a GRADE-informed approach considering risk of bias, inconsistency, indirectness, imprecision, and publication bias [13].

Synthesis Methods

A quantitative meta-analysis was considered only if the eligible controlled studies were sufficiently homogeneous in their interventions, comparators, follow-up durations, outcome definitions, and statistical reporting. Meta-analysis was not performed because the controlled studies differed substantially in intervention type, including mandibular advancement appliances, oral jaw-positioning appliances, and rapid, semi-rapid, or slow maxillary expansion; comparator type, including untreated controls, sham appliances, and adenotonsillectomy; follow-up duration; and outcome reporting, including apnea index, apnea-hypopnea index, percentage change, and effect estimates with confidence intervals.

Only one controlled study provided a pool-compatible effect estimate with a retrievable variance measure. Pooling the remaining studies would have required unsupported assumptions or imputation of variance data, which was not considered methodologically appropriate. Therefore, findings were synthesized narratively by intervention class, and existing published meta-analyses were cited as external quantitative benchmarks where relevant. No pooled estimate, forest plot, or funnel plot was generated because doing so would risk conveying false precision.

Results

Study Selection

**Figure 1:**
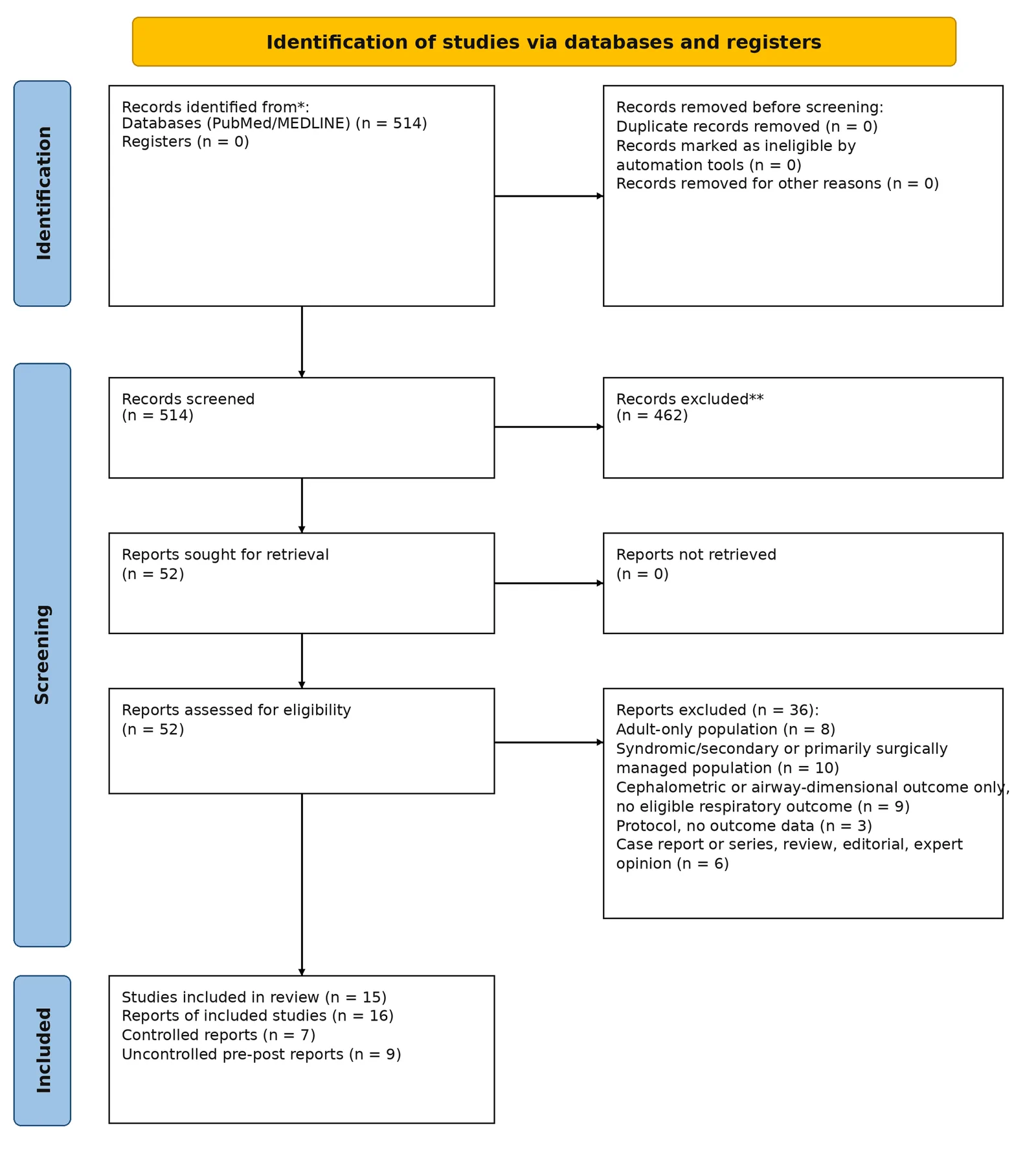
PRISMA 2020 flow diagram of study selection. The search retrieved 728 records before application of the predefined English-language and pediatric-age database limits. Application of these limits removed 54 non-English records and 160 records outside the pediatric age criteria, leaving 514 records for screening. Because only PubMed/MEDLINE was searched, no duplicate records were identified or removed. After title and abstract screening, 52 reports underwent full-text assessment; 16 reports, representing 15 unique studies, met the eligibility criteria. Two reports from one partially overlapping study cohort were retained as separate reports but counted as one study in the overall evidence totals. Quantitative meta-analysis was not performed because of clinical and methodological heterogeneity and incomplete extractable variance data. PRISMA: Preferred Reporting Items for Systematic Reviews and Meta-Analyses [9]

The search retrieved 728 records before application of the predefined English-language and pediatric-age database limits. The English-language limit removed 54 records, and the pediatric-age limit removed 160; 514 records remained and were identified for screening (Figure 1). We removed no duplicate records because we searched a single database. After title and abstract screening, 462 records were excluded, and 52 reports were sought for retrieval; all were obtained and assessed in full text. Of these, 36 were excluded because they involved adult-only populations, syndromic or surgically managed secondary populations, cephalometric or airway-dimensional outcomes without respiratory endpoints, protocols without outcome data, or case reports, reviews, editorials, or expert-opinion articles.

Sixteen reports representing 15 unique studies were included [14-29]: seven controlled reports and nine uncontrolled pre-post reports arising from eight cohorts (Figure 1; Table 3). Villa et al. 2007 [23] and Villa et al. 2015 [24] were identified as two reports from one partially overlapping study cohort. The 2015 publication explicitly continued the earlier protocol and added 26 newly completed participants to the 14 completers from the 2007 report, yielding a combined cohort of 40. Accordingly, the publications were retained as separate reports but counted as one study in the overall evidence totals. Pirelli et al. 2015 [30] was used as a contextual follow-up citation and was not treated as an included report in the review dataset; it therefore does not appear in the PRISMA included-report count.

**Table 3:** Characteristics and Respiratory Outcomes of Included Interventional Reports AHI, apnea-hypopnea index; OSA, obstructive sleep apnea; PSG, polysomnography; RCT, randomized controlled trial; RME, rapid maxillary expansion; SaO₂/SpO₂, arterial oxygen saturation; SEM, standard error of the mean; SRME, semi-rapid maxillary expansion; AT, adenotonsillectomy; RPE, rapid palatal expansion ᵃ The publication reports inconsistent group mean ages; no pooled value was calculated. ᵇ No concurrent comparator for respiratory outcomes; classified as uncontrolled. ᶜ Participants were randomized to treatment sequence. First-period outcomes are summarized; second-period results were treated as supportive longitudinal evidence. ᵈ Healthy comparison subjects received no intervention or repeat polysomnography. Cohort overlap: Villa et al. 2007 [23] reported 14 participants who completed the original 12-month trial. Villa et al. 2015 [24] explicitly continued that protocol and added 26 newly completed participants, yielding a combined cohort of 40. The publications were retained as separate reports because they provide different analyses and follow-up information but were counted as one study. Outcomes unique to each report were extracted separately, while overlapping participants were not double-counted in overall study totals, narrative synthesis, or certainty assessments.

Report	Design	Intervention	Comparator	N	Age (years)	Respiratory outcome
Villa 2002 [14]	RCT (parallel group)	Oral jaw-positioning appliance	Untreated control	32 randomized; 23 analyzed	7.1 ± 2.6 (all randomized)	Apnea and hypopnea indices were significantly lower after 6 months in the treated group; controls were essentially unchanged.
Machado-Júnior 2016 [15]	Pilot RCT (parallel group)	Mandibular advancement appliance	No treatment	16 randomized; 14 analyzed	Mean ages reported inconsistently between the text and table ᵃ	AHI decreased in the treated group and increased in controls at 12 months.
Idris 2018 [16]	Randomized crossover trial (sham-controlled; 3-week periods, 2-week washout)	Mandibular advancement splint	Sham splint	18 randomized; 16 analyzed	9.8 ± 1.4 (analyzed)	Overall AHI decreased by 37% (95% CI, 15%-53%; p=0.002); supine AHI decreased by 4.1 events/h (95% CI, 1.8-6.4; p<0.001).
Zreaqat 2023 [17]	Uncontrolled pre-post study for the respiratory outcome ᵇ	Twin-block appliance	Pre-treatment baseline	34 treated	10.29 ± 1.21	AHI decreased by 11.2 ± 4.6 events/h, a 74.8% reduction (p<0.001), within the treated group only.
Magalhães 2024 [18]	RCT (parallel-group comparison of treatment sequences) ᶜ	Rapid palatal expansion first	Adenotonsillectomy first	32 randomized; 30 completed both phases	8.9 (AT-first); 8.1 (RPE-first)	After the first assigned intervention, AHI decreased significantly from baseline in the adenotonsillectomy-first group, whereas the rapid-palatal-expansion-first group did not show a statistically significant reduction until the subsequent treatment phase. The reported p=0.035 pertains to the end-of-trial comparison, not to the first-period comparison.
Hoxha 2018 [19]	RCT (parallel group); allocation method not described	Semi-rapid maxillary expansion	Untreated control	30 (15 per group)	Control 11.46 ± 2.06; SRME 12.27 ± 1.93	AHI decreased significantly in both groups (control 2.67 ± 1.23 to 1.80 ± 1.08; treated 2.50 ± 1.12 to 1.79 ± 1.05); the between-group difference was not statistically significant.
Aksilp 2025 [20]	RCT (parallel group)	Rapid maxillary expansion	Adenotonsillectomy	24	6.33 ± 1.71	Both interventions improved AHI at 6 months; no significant between-group difference in PSG parameters or cure rate, while symptom and quality-of-life improvement favored adenotonsillectomy.
Guilleminault 2011 [21]	Pilot RCT (parallel-group comparison of treatment sequences) ᶜ	Adenotonsillectomy first	Rapid maxillary distraction first	31 randomized; 30 completed both phases	6.5 ± 0.2 (SEM)	After the first assigned intervention, both strategies improved AHI with no significant difference by order; only one child normalized after the first intervention alone.
Pirelli 2004 [22]	Uncontrolled pre-post study	RME	Baseline	31	8.68 (range 6-12)	Mean AHI decreased from 12.18 ± 2.6 to 0.4 ± 1.1 events/h; nadir SpO₂ rose from 78.5 ± 8.2% to 95.3 ± 1.7%.
Villa 2007 [23]	Uncontrolled pre-post study	RME	Baseline	16 enrolled; 14 analyzed	6.9 ± 2.2 (enrolled); 6.6 ± 2.0 (analyzed)	AHI decreased significantly at 12 months; mean SaO₂ was not significantly changed.
Villa 2015 [24]	Uncontrolled pre-post study	RME	Baseline	40	6.3 ± 1.6 (range 4.3-10.5)	Thirty-four of 40 children were classified as responders; residual OSA persisted in 57.5%.
Buccheri 2017 [25]	Uncontrolled pre-post study	RME	Baseline	11	6.9 ± 1.04	AHI decreased from 6.09 ± 3.47 to 2.36 ± 2.24 events/h; oxygen saturation increased from 93.1% to 96.8%.
Zreaqat 2024 [26]	Uncontrolled pre-post study	RME	Baseline	34	10.29	AHI decreased by 5.43 events/h; lowest SpO₂ increased by 5.62% (p<0.001); oxygen desaturation index decreased by 5.76 events/h (p=0.024).
Kushida 2025 [27]	Multicenter uncontrolled pre-post study	Non-permanent orthodontic intraoral device / slow maxillary expansion	Baseline	55 enrolled; 47 analyzed	Mean age not reported; eligibility <18 y	AHI improved in 79% of participants; 61.7% improved by ≥50%, and 17% had OSA resolution, with follow-up up to 24 months.
Cozza 2004 [28]	Uncontrolled pre-post study ᵈ	Modified monobloc	Baseline	20 treated	5.91 (range 4-8)	Median AHI decreased after treatment; minimum SaO₂ was not significantly changed (97.39% to 96.87%, p=0.407).
Kiran Aydil 2026 [29]	Uncontrolled pre-post study with 7-year follow-up	Monobloc mandibular advancement	Baseline	16 initial cohort; 13 analyzed at 7 years	10.97 ± 1.51 at treatment initiation	AHI decreased from 2.3 (1-16.9) to 0.5 (0.2-1.8) after treatment and returned to 2 (0.1-26) at 7 years (p=0.002); mean oxygen saturation declined significantly at 7 years (97.46 ± 0.78% to 96.23 ± 1.36%, p=0.002).

Characteristics of Included Studies

The included studies were conducted across Europe, the Americas, and Asia between 2002 and 2026. Sample sizes were small, with 11 to 55 participants enrolled and 13 to 47 analyzed. Interventions included maxillary expansion (rapid, semi-rapid, and slow); mandibular advancement or functional appliances (oral jaw-positioning appliances, mandibular advancement splints, twin-block appliances, and monobloc therapy); and combined orthodontic-adenotonsillectomy sequencing. Comparators included untreated controls, sham appliances, adenotonsillectomy, or pre-treatment baseline. All reports presented the apnea-hypopnea index or a closely related polysomnographic respiratory index. Numeric oxygen-saturation or desaturation outcomes were reported in 12 reports representing 11 unique studies or cohorts [16,18-26,28,29]. Seven reports described significant improvement [16,20,21,24-26], four reported no significant change [18,19,23,28], and one reported a significant decline in mean oxygen saturation at seven-year follow-up [29].

The seven controlled reports were described by their authors as randomized trials, although the adequacy and reporting of randomization varied substantially and one study used predictable alphabetical allocation [14].

Synthesis: Mandibular Advancement and Functional Appliances

The most directly informative controlled evidence concerned mandibular advancement and functional appliances. In a randomized trial of an oral jaw-positioning appliance versus an untreated control in 32 children with malocclusion and a baseline apnea index greater than one event per hour, treated children showed significantly lower apnea and hypopnea indices after six months, while controls were essentially unchanged [14]; allocation in that trial was made alphabetically by surname, and it is assessed at high risk of bias. A pilot randomized trial of a mandibular advancement appliance versus no treatment randomized 16 children, of whom 14 were analyzed, and reported a significant decrease in the apnea-hypopnea index in the treated arm with an increase in controls over 12 months [15]. A sham-controlled crossover randomized trial of a mandibular advancement splint in 18 children with SDB found a 37% reduction in overall apnea-hypopnea index (95% CI, 15%-53%; p=0.002) and a reduction in supine apnea-hypopnea index of 4.1 events/h (95% CI, 1.8-6.4; p<0.001) [16]. The sham mandibular advancement splint consisted of removable upper and lower acrylic plates that resembled the active Twin-Block appliance but contained no component designed to advance the mandible. It served as the control condition; however, the investigators noted that it was not completely physiologically inert because it could still influence mandibular and tongue posture [16]. These findings are directionally consistent with an existing network meta-analysis that estimated a modest pooled reduction in the apnea-hypopnea index for mandibular advancement appliances versus control of approximately 2.2 events/h (95% CI, -3.48 to -0.89) [31], as well as a recent twin-block meta-analysis [32]. However, an earlier systematic review of mandibular advancement appliances assessed the underlying evidence as being at high risk of bias [33]. In addition, functional appliances used for Class II correction produce skeletal and dentoalveolar changes that should be considered when interpreting occlusal and potential airway-related responses in growing patients, as summarized by Almrayati et al. [34].

Uncontrolled evidence for this intervention class was more limited. An uncontrolled pre-post study of twin-block therapy in 34 children reported a within-group reduction in the apnea-hypopnea index of 11.2 (SD 4.6) events/h, a 74.8% decrease (p<0.001) [17]; the comparison group in that study comprised children without OSA who did not undergo repeat polysomnography, so it provides no controlled respiratory comparison. Monobloc functional therapy in young children with OSA has been reported to reduce polysomnographic indices [28], with one longitudinal study documenting initial improvement in the apnea-hypopnea index followed by partial recurrence during adolescence and a significant decline in mean oxygen saturation at seven-year follow-up, emphasizing the modifying role of growth over time [29].

Synthesis: Maxillary Expansion

Maxillary expansion represented the largest body of pediatric evidence, but the predominance of uncontrolled pre-post designs limited the certainty of this evidence. The index cohort reported a fall in the mean apnea-hypopnea index from 12.18±2.6 to 0.4±1.1 events/h after rapid maxillary expansion in 31 children [22], with a subsequent report describing sustained benefit at long-term follow-up in the same cohort [30]. This cohort overlap precluded independent pooling. Additional uncontrolled series reported significant reductions in the apnea-hypopnea index after rapid maxillary expansion [23-26] and after slow or serial expansion in a multicenter pre-post study of 55 enrolled participants, of whom 47 were analyzed [27]. Three-dimensional documentation of morphological skeletal and dentoalveolar variations between rapid and slow maxillary expansion modalities in growing patients has been reported [35], further illustrating the clinical heterogeneity among expansion protocols. Two of these reports by Villa et al. [23,24] describe one partially overlapping cohort.

Controlled data were less favorable than the uncontrolled series suggest. In the only included trial with an untreated concurrent control, the apnea-hypopnea index fell significantly in both the semi-rapid maxillary expansion group (2.50±1.12 to 1.79±1.05) and the untreated control group (2.67±1.23 to 1.80±1.08) over five months, and the between-group difference was not statistically significant [19]. This within-review finding directly illustrates that within-group improvement in uncontrolled series may reflect spontaneous change rather than a treatment effect. In a randomized comparison of rapid palatal expansion with adenotonsillectomy as the first intervention in 32 non-obese children with tonsillar hypertrophy and maxillary constriction, the apnea-hypopnea index fell significantly from baseline in the adenotonsillectomy-first group after the first assigned intervention. In contrast, the expansion-first group did not show a statistically significant reduction until the subsequent treatment phase [18]. A further randomized trial comparing rapid maxillary expansion with adenotonsillectomy in 24 children found that both interventions improved the apnea-hypopnea index at six months with no significant between-group difference in polysomnographic parameters or cure rate, while symptom and quality-of-life improvement favored adenotonsillectomy [20]. A pilot randomized trial comparing two treatment strategies, adenotonsillectomy first versus rapid maxillary distraction first, in 31 children found that only one child normalized after the first intervention alone and that treatment order did not significantly affect the degree of improvement [21]; all children ultimately received both interventions.

Consistent with these controlled findings, a systematic review restricted to controlled evidence found only one randomized comparison of rapid maxillary expansion against watchful waiting, which showed no statistically significant difference in the apnea-hypopnea index, highlighting spontaneous resolution as a major confounder in the uncontrolled literature [36]. National pediatric guidance has likewise concluded that the evidence is insufficient to recommend rapid maxillary expansion as the primary therapy for pediatric OSA [1], and a focused review of expansion-plus-mandibular-advancement appliances reached similarly cautious conclusions [37].

Synthesis: Combined Approaches and Adjunctive Therapies

Combined or sequenced approaches involving orthodontic therapy and adenotonsillectomy suggest potential additive anatomical effects but do not establish that orthodontic intervention can substitute for first-line medical therapy [18,21]. Because the two trials that evaluated sequencing delivered both interventions to every participant without a washout, their end-of-trial results describe the cumulative effect of two treatments rather than the isolated effect of either. They are reported here as supportive longitudinal evidence only. Myofunctional therapy, frequently discussed as an orthodontic or dental adjunct, has only low-certainty support in children according to Cochrane appraisal [38].

Why Meta-Analysis Was Not Performed

We did not perform a quantitative meta-analysis because the controlled studies were too clinically and methodologically heterogeneous to pool responsibly. The eligible studies evaluated different interventions, including mandibular advancement appliances, oral jaw-positioning appliances, and rapid, semi-rapid or slow maxillary expansion; used different comparators, including untreated controls, sham appliances, and adenotonsillectomy; had follow-up periods ranging from three weeks per treatment period to seven years; and reported outcomes using incompatible formats, including apnea index, apnea-hypopnea index, percentage change, or effect estimates with confidence intervals. Only one controlled study [16] provided a pool-compatible effect estimate with a retrievable variance measure. Pooling the remaining studies would have required unsupported assumptions or imputation of variance data, which we did not consider methodologically appropriate. Existing meta-analyses that pooled selected subsets of the literature [31,32,37] are therefore cited as external quantitative benchmarks rather than re-derived. Accordingly, we present no pooled estimate, forest plot, or funnel plot, because doing so would risk conveying false precision.

Risk of Bias and Certainty of Evidence

Risk-of-bias and quality assessments are presented separately by study design in Tables 4-6 [10-12], because the applicable instruments use different domains and different judgment categories and are not directly interconvertible. Outcome-level certainty judgments derived from the GRADE-informed appraisal are summarized in Table 7 [13].

**Table 4:** Risk-of-Bias Assessment of Randomized Parallel-Group Trials Using RoB 2 RoB 2 was applied to individually randomized parallel-group trials [10]. Judgments are presented as Low risk of bias, Some concerns, or High risk of bias for the apnea-hypopnea index or the equivalent respiratory outcome evaluated in this review. RoB 2 assesses the risk of bias in a specific trial result rather than assigning an overall methodological-quality score to a publication. Magalhães 2024 [18] and Guilleminault 2011 [21] randomized participants to treatment sequences involving irreversible or persistent interventions. Because complete washout was impossible, risk-of-bias judgments were based on the randomized comparison after the first assigned intervention; second-period results were treated as supportive longitudinal evidence only.

Study	D1: Randomization process	D2: Deviations from intended interventions	D3: Missing outcome data	D4: Measurement of the outcome	D5: Selection of the reported result	Overall risk of bias
Villa 2002 [14]	High	Some concerns	Some concerns	Some concerns	Some concerns	High
Machado-Júnior 2016 [15]	Some concerns	Some concerns	Some concerns	Low	Some concerns	Some concerns
Magalhães 2024 [18]	Low	Low	Low	Low	Some concerns	Some concerns
Hoxha 2018 [19]	Some concerns	Some concerns	Low	Some concerns	Some concerns	Some concerns
Aksilp 2025 [20]	Some concerns	Low	Low	Some concerns	Low	Some concerns
Guilleminault 2011 [21]	High	Some concerns	Low	Some concerns	Some concerns	High

**Table 5:** Risk-of-Bias Assessment of the Randomized Crossover Trial Using the RoB 2 Tool for Crossover Trials Idris 2018 [16] was the only included trial assessed with the RoB 2 extension for crossover trials [11], which adds Domain S (bias arising from period and carryover effects) to the five standard RoB 2 domains. Judgments are presented as Low risk of bias, Some concerns, or High risk of bias for the apnea-hypopnea index or the equivalent respiratory outcome evaluated in this review. Under the RoB 2 algorithm, an overall judgment of Low risk of bias requires all domains, including Domain S, to be rated low. Domain S was judged Low risk of bias because a two-week washout separated removable active and sham mandibular advancement splints, the analysis accounted for period effects, and no evidence of carryover was detected. The two sequence-randomized trials with irreversible or persistent interventions [18,21] were assessed as randomized parallel-group comparisons in Table 4.

Study	D1: Randomization process	DS: Period and carryover effects	D2: Deviations from intended interventions	D3: Missing outcome data	D4: Measurement of the outcome	D5: Selection of the reported result	Overall risk of bias
Idris 2018 [16]	Low	Low	Low	Some concerns	Low	Low	Some concerns

**Table 6:** Quality Assessment of Uncontrolled Before-After (Pre-Post) Studies Using the NHLBI Before-After Tool Uncontrolled before-after studies were appraised with the National Heart, Lung, and Blood Institute (NHLBI) Quality Assessment Tool for Before-After (Pre-Post) Studies With No Control Group [12]. Each criterion is rated “Yes”, “No”, “Cannot determine” (CD), “Not reported” (NR) or “Not applicable” (NA); the overall rating is “Good”, “Fair” or “Poor”. The NHLBI states that these tools have not been independently published and are not considered standardized, so ratings are reported as structured quality judgments rather than validated risk-of-bias estimates. Criterion 12 is not applicable throughout because every included study delivered the intervention at the individual rather than the group level. Zreaqat 2023 [17] appears in this table rather than among the controlled studies because its comparison group comprised children without obstructive sleep apnea who did not undergo repeat polysomnography. Villa 2007 [23] and Villa 2015 [24] are appraised separately but counted once in all evidence totals. Criterion 4 was rated No for both because only 16 of 35 and 30 of 64 eligible children, respectively, agreed to participate. Villa 2015 [24] and Kushida 2025 [27] were the only reports to document blinded outcome assessment. Kushida 2025 [27] is the only report rated Good; it was registered retrospectively (ClinicalTrials.gov NCT05661747), and two of its authors are affiliated with the device sponsor, both of which readers should weigh. Buccheri 2017 [25] is rated Poor because it enrolled 11 participants without formal eligibility criteria, a sample-size calculation, attrition reporting or blinded assessment, and measured the outcome at only two time points.

Criterion	Zreaqat 2023 [17]	Pirelli 2004 [22]	Villa 2007 [23]	Villa 2015 [24]	Buccheri 2017 [25]	Zreaqat 2024 [26]	Kushida 2025 [27]	Cozza 2004 [28]	Kiran Aydil 2026 [29]
1. Study question or objective clearly stated	Yes	Yes	Yes	Yes	Yes	Yes	Yes	Yes	Yes
2. Eligibility/selection criteria prespecified and clearly described	Yes	Yes	Yes	Yes	CD	Yes	Yes	Yes	Yes
3. Participants representative of those eligible for the intervention	Yes	Yes	No	No	CD	Yes	Yes	CD	Yes
4. All eligible participants meeting entry criteria enrolled	CD	Yes	No	No	CD	CD	CD	CD	Yes
5. Sample size sufficiently large to provide confidence in the findings	Yes	No	No	No	No	No	Yes	No	No
6. Intervention clearly described and delivered consistently	Yes	Yes	Yes	Yes	Yes	Yes	Yes	Yes	Yes
7. Outcome measures prespecified, clearly defined, valid, reliable, consistently assessed	Yes	Yes	Yes	Yes	Yes	Yes	Yes	Yes	Yes
8. Outcome assessors blinded to participants' interventions	NR	NR	NR	Yes	NR	NR	Yes	NR	NR
9. Loss to follow-up ≤20% and those lost accounted for in the analysis	CD	Yes	Yes	Yes	CD	CD	Yes	CD	Yes
10. Statistical methods examined pre-to-post change with p values	Yes	Yes	Yes	Yes	Yes	Yes	Yes	Yes	Yes
11. Outcomes measured multiple times before and after the intervention	No	Yes	Yes	Yes	No	No	Yes	No	Yes
12. Group-level intervention analyzed using individual-level data	NA	NA	NA	NA	NA	NA	NA	NA	NA
Overall quality rating	Fair	Fair	Fair	Fair	Poor	Fair	Good	Fair	Fair

**Table 7:** GRADE-Informed Certainty Appraisal by Outcome AHI, apnea-hypopnea index; GRADE, Grading of Recommendations Assessment, Development and Evaluation [13]; SDB, sleep-disordered breathing. The four AHI rows account for all 16 included reports and all 15 unique studies or cohorts without double counting (3+4+6+3 = 16 reports; 3+4+5+3 = 15 unique studies). The oxygen-saturation row is deliberately not additive with the AHI rows, because most reports contribute to both an apnea-hypopnea index outcome and a saturation outcome. Villa 2007 [23] and Villa 2015 [24] are counted as one cohort in every unique-studies column. A report was counted in the oxygen-saturation row only where it presented a numeric oxygen-saturation or desaturation result relevant to treatment response; Machado-Júnior 2016 [15] and Kushida 2025 [27] report no saturation data, Zreaqat 2023 [17] mentions saturation only incidentally, and Villa 2002 [14] states narratively that the desaturation index decreased without significance but reports no numeric value. Certainty judgments represent a structured application of GRADE domains and were not independently duplicated or developed as a formal guideline evidence profile.

Outcome / comparison	Reports (n)	Unique studies/cohorts (n)	Contributing references	Key GRADE considerations	Finding	Certainty
AHI - mandibular advancement / functional appliances versus untreated or sham control	3	3	[14-16]	Downgraded for risk of bias (one trial at high risk) and for imprecision from very small samples	AHI decreased in treated groups; the sham-controlled crossover trial reported a 37% reduction in overall AHI [16], directionally consistent with a network meta-analytic estimate of approximately -2.2 events/h [31]	Low
AHI - maxillary expansion versus untreated control, watchful waiting, or adenotonsillectomy	4	4	[18-21]	Downgraded for risk of bias, imprecision, indirectness (two trials compare expansion against surgery rather than against no treatment), and confounding by spontaneous resolution	Controlled evidence does not support expansion as primary therapy. In the only trial with an untreated concurrent control, AHI fell comparably in both arms with no significant between-group difference [19]; adenotonsillectomy produced a significant first-period reduction where expansion did not [18], and the two were equivalent in another trial [20]; an external controlled-evidence review found no significant AHI difference versus watchful waiting [36]	Very low
AHI - maxillary expansion in uncontrolled pre-post studies	6	5	[22-27]	Starts at low certainty; further downgraded for serious risk of bias, absence of any control group, and confounding by growth and spontaneous resolution	Large within-group AHI reductions were reported, but the magnitude of benefit is likely overstated	Very low
AHI - mandibular advancement / functional appliances in uncontrolled pre-post studies	3	3	[17,28,29]	Starts at low certainty; further downgraded for serious risk of bias, absence of a control group, and confounding by growth	Within-group AHI reductions were reported, with return toward baseline at seven-year follow-up in one study [29]	Very low
Oxygen saturation or desaturation	12	11	[16,18-26,28,29]	Downgraded for risk of bias, imprecision, and inconsistency of both direction and metric, since mean saturation, nadir saturation, and desaturation index are not interchangeable	Findings are inconsistent. Significant improvement was reported in [16,20-24,26]; no significant change in [18,19,23,28]; and a significant decline in mean saturation at seven-year follow-up in [29]	Very low
Prevention of future SDB	0	0	-	No direct evidence identified	No included report addresses prevention of future SDB; current guidance does not support preventive claims [4]	Insufficient evidence

Among the randomized parallel-group trials (Table 4), two were judged at high risk of bias. In Villa et al. 2002 [14], participants were assigned “alphabetically, by surname,” which is neither a random sequence nor concealable and produced unequal groups. In Guilleminault et al. 2011 [21], a pre-established randomization table was used in pairs, with every second allocation determined automatically, so allocation concealment could not be achieved. The remaining four parallel-group trials [15,18,19,20] were rated as raising some concerns, most often because allocation concealment was insufficiently described, polysomnographic scoring was unblinded, or no protocol or prospective registration was available. Magalhães et al. 2024 [18] and Aksilp et al. 2025 [20] were the best reported, with concealed allocation and blinded outcome measurement, respectively.

The single randomized crossover trial (Table 5), Idris et al. 2018 [16], provided the most methodologically robust evidence in the review: it was prospectively registered with a published protocol, used concealed block randomization, blinded participants to active versus sham appliance, incorporated a two-week washout, and formally tested for carryover effects, finding none. Its overall rating is nevertheless “some concerns” rather than “low risk of bias,” because the numbers of participants randomized, withdrawn, and analyzed reported in the paper do not reconcile.

The nine uncontrolled before-after reports (Table 6) were rated one Good, seven Fair, and one Poor. They were limited principally by the absence of a concurrent control group, which precludes separation of treatment effects from growth, regression to the mean, concurrent care, and spontaneous improvement, and additionally by the absence of a priori sample-size calculation in seven of the nine, by unreported blinding of outcome assessment in seven of the nine, and by unreported attrition in four.

Using a structured application of GRADE domains, confidence in the estimated effects of orthodontic interventions on the apnea-hypopnea index and on oxygen saturation was judged to be low to very low across intervention classes. Downgrading reflected risk of bias, imprecision, inconsistency, and, where the available comparator did not directly address the review question, indirectness.

Discussion

Principal Findings

Within a heterogeneous, mostly low-certainty evidence base, selected orthodontic interventions were associated with reductions in the apnea-hypopnea index in appropriately selected, medically diagnosed children. The comparatively stronger controlled evidence concerned mandibular advancement appliances, where a sham-controlled randomized crossover trial converges with a published network meta-analytic estimate of modest benefit [16,31]; even that trial, however, was rated as raising some concerns, and the two parallel-group trials in the same intervention class were rated high risk and some concerns, respectively [14,15].

For maxillary expansion, the picture is weaker than the uncontrolled literature implies. The apparent benefit rested largely on uncontrolled pre-post studies. At the same time, the controlled evidence within this review found no significant advantage over an untreated control [19], no significant advantage over adenotonsillectomy [20], and a slower initial response than adenotonsillectomy when expansion was performed first [18]. This is consistent with an external controlled-evidence review that found no statistically significant difference against watchful waiting [36]. Spontaneous resolution is therefore a major confounder, and the finding that untreated children improved as much as treated children over five months [19] should temper interpretation of every uncontrolled series in this field.

Neither any included study nor any current professional guideline supports the claim that orthodontic treatment prevents future SDB [4].

Integration With Professional Guidance

These findings align closely with current professional guidance. The AAO 2019 White Paper and 2026 update define the orthodontist’s role as screening and referral, not diagnosis [3,4]. The 2026 update further emphasizes that current evidence does not support claims that orthodontic intervention prevents SDB, or that CBCT or lateral cephalograms are valid screening or diagnostic tools for SDB [4]. The AAPD similarly frames the dental team’s role as identifying risks and making timely medical referrals, with orthodontic assessment considered before appliance therapy when appropriate [6]. AADSM/AASM guidance on oral appliance therapy is primarily adult-oriented and does not establish a standardized pediatric orthodontic protocol [7]. The AAOP perspective places sleep bruxism and orofacial pain within an interdisciplinary care model, reinforcing that the orthodontist or orofacial pain clinician functions as one member of a physician-led team rather than as an independent manager of SDB [8].

Orofacial pain specialists may contribute meaningfully to interdisciplinary care when temporomandibular disorders, sleep bruxism, headache, or other orofacial pain symptoms coexist with pediatric SDB. Some orofacial pain specialists also have additional training or board qualification in dental sleep medicine, including ABDSM qualification or diplomate status. However, dental sleep medicine training varies among board-certified orofacial pain specialists. Therefore, referral or co-management should be based on the clinician’s relevant sleep medicine training, scope of practice, and ability to collaborate within a physician-led sleep team, rather than on orofacial pain board certification alone.

Three clinical principles follow from this evidence and guidance: orthodontists screen and refer; physicians diagnose; and polysomnography remains the diagnostic reference standard. Screening tools appropriate to the orthodontic setting include validated questionnaires such as the Pediatric Sleep Questionnaire, structured history, and clinical examination, including tonsil grading, modified Mallampati assessment, and craniofacial evaluation [39]. These tools identify risk and guide referral, but they do not establish a diagnosis. Imaging acquired for orthodontic purposes should not be repurposed for SDB screening or diagnosis [4]; such images capture static, awake anatomy and are not validated as diagnostic tests for SDB.

Clinical Implications and a Guideline-Anchored Framework

**Figure 2:**
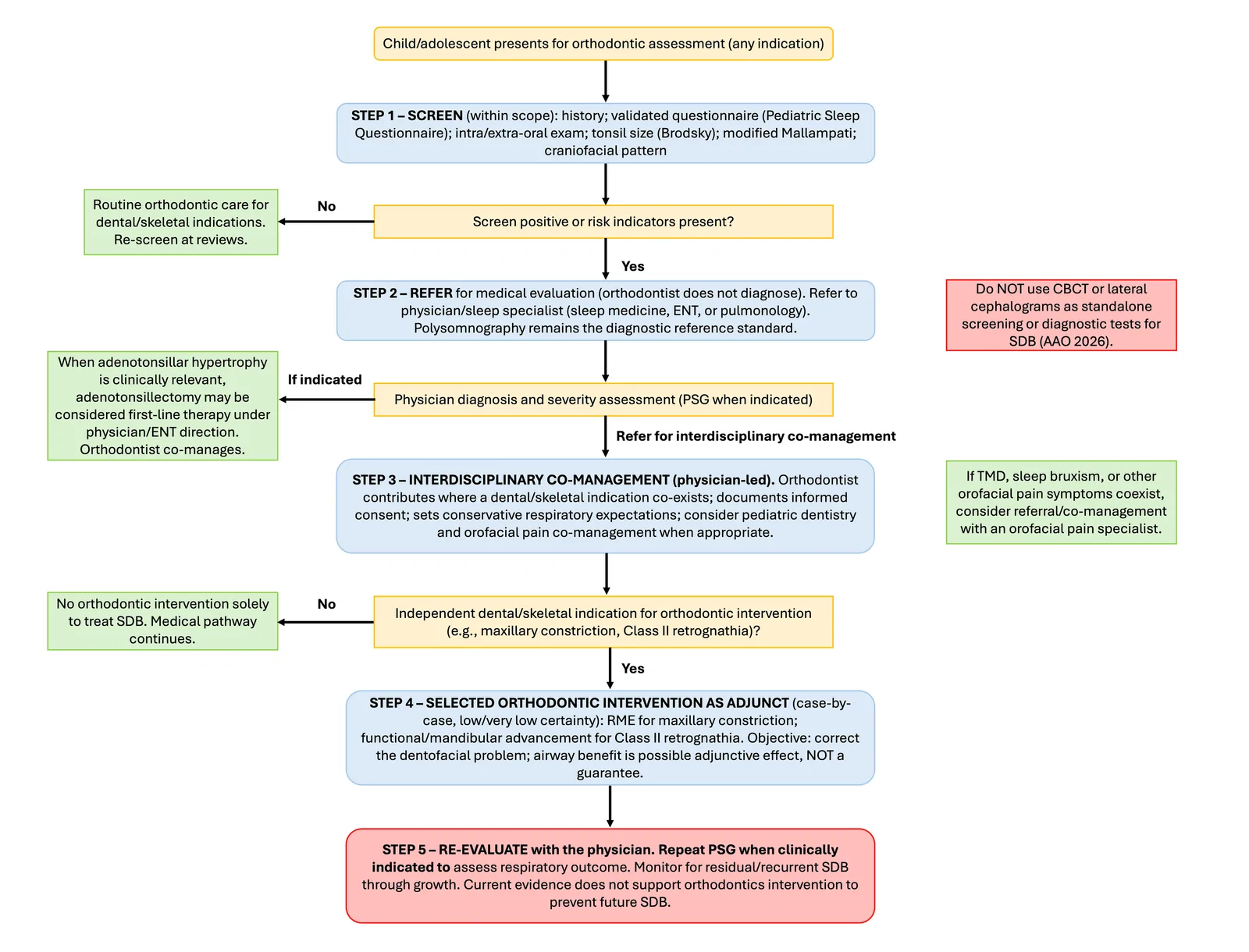
Clinical Decision Framework for the Orthodontist in Pediatric Sleep-Disordered Breathing (SDB) The framework emphasizes risk identification and referral for physician-led diagnosis and management, with orthodontic intervention considered only as an adjunct when an independent dentofacial indication is present. Cone-beam computed tomography (CBCT) and lateral cephalometric imaging are not recommended as standalone screening or diagnostic tests for pediatric SDB. The framework is informed by current American Association of Orthodontists (AAO) and American Academy of Pediatric Dentistry (AAPD) recommendations and, where applicable, American Academy of Dental Sleep Medicine (AADSM)/American Academy of Sleep Medicine (AASM) guidance and American Academy of Orofacial Pain (AAOP) perspectives on interdisciplinary care. PSG, polysomnography; TMD, temporomandibular disorder; RME, rapid maxillary expansion Image credit: Created by the authors using Microsoft PowerPoint (Redmond, WA, USA).

To operationalize this evidence and guidance, we propose a stepwise clinical decision framework (Figure 2). The framework positions the orthodontist as a screener and interdisciplinary contributor: validated screening during orthodontic assessment; referral for medical evaluation when risk indicators are identified; physician-led diagnosis and management; and, within the conservative clinical framework proposed in this review, consideration of orthodontic intervention for SDB when an independent dental or skeletal indication for orthodontic treatment also exists. This recommendation represents the authors’ synthesis and clinical interpretation of the evidence and professional guidance reviewed here, rather than a specific recommendation from a single guideline. When pediatric OSA/SDB is confirmed, and clinically significant adenotonsillar hypertrophy is present, the physician or ENT may consider adenotonsillectomy, provided there are no contraindications to surgery. Other physician-directed management may include weight management, nasal or allergy management, medical therapy, or continuous positive airway pressure (CPAP) for persistent or severe disease.

Orthodontic intervention should be framed to the family as treatment of the dentofacial problem, with airway benefit as a possible adjunctive effect rather than a guaranteed or preventive outcome, and conservative respiratory expectations should be documented in the informed consent record. Re-evaluation with the physician, including repeat polysomnography when clinically indicated, closes the loop and helps monitor residual or recurrent disease through growth. This framework deliberately incorporates the 2026 AAO cautions against imaging-based diagnosis and preventive claims [4].

The framework is guided by the principle of “first, do no harm.” For orthodontists, this means identifying risk without diagnosing SDB, referring promptly for medical evaluation, avoiding CBCT- or cephalogram-based diagnostic claims, and, within the framework proposed here, reserving orthodontic intervention for patients with an independent dental or skeletal indication for treatment. Orthodontic care should therefore remain adjunctive to, rather than substituted for, physician-led management.

Strengths and Limitations

Strengths of this review include explicit PICOS-based eligibility criteria, study selection and data extraction involving three reviewers, transparent separation of controlled and uncontrolled evidence based on whether a concurrent comparator contributed respiratory data, design-specific risk-of-bias and quality appraisal with a GRADE-informed certainty rating, explicit handling of overlapping cohorts, integration of current multi-society guidance including the 2026 AAO update, and a deliberate decision not to pool clinically heterogeneous studies when doing so could create false precision.

Several limitations should be acknowledged. First, the search was limited to PubMed/MEDLINE and may therefore have missed eligible studies indexed exclusively in other bibliographic databases, grey-literature sources, or trial registries. The absence of grey-literature and trial-registry searching may also have increased susceptibility to publication bias. Second, we included only English-language publications because resources for reliable translation and verification of non-English full-text reports were not available. This restriction excluded 54 records at the database-search stage and may have introduced language bias, particularly given the geographically diverse evidence base. Third, risk-of-bias judgments were based on information available in the published reports and may have been affected by incomplete reporting. Fourth, the underlying evidence base was dominated by small studies, short follow-up periods, heterogeneous interventions and comparators, and uncontrolled pre-post designs vulnerable to confounding by growth, regression to the mean, concurrent care, and spontaneous improvement. Fifth, the review was not prospectively registered, and no protocol was published, limiting readers’ ability to independently verify whether the review methods and analyses were specified a priori. Sixth, the uncontrolled before-after studies were appraised using the NHLBI quality-assessment tool, which its developers state has not been independently published or standardized; those ratings should therefore be interpreted as structured quality judgments rather than as validated risk-of-bias estimates. Seventh, Villa et al. 2007 [23] and Villa et al. 2015 [24] reported a partially overlapping cohort. Although the publications were treated as one study and overlapping participants were not counted twice in review-level totals, some participants contributed outcome data reported across both publications. Eighth, potential participant overlap between the reports by Zreaqat et al. published in 2023 [17] and 2024 [26] could not be ruled out because of similarities and internal inconsistencies in their reported demographic data. The reports were nevertheless counted as separate studies because participant overlap could not be confirmed and they evaluated different interventions in differently defined populations.

These limitations constrain the strength of causal inference and are reflected in the low-to-very-low certainty judgments. The certainty appraisal was informed by GRADE domains but was not developed as a formal GRADE evidence profile; the ratings should therefore be interpreted as review-level judgments rather than definitive guideline-level determinations.

Future Research

Future studies should include adequately powered randomized trials with concurrent untreated, sham, or watchful-waiting controls; standardized polysomnographic outcome reporting with complete variance data; longer follow-up spanning growth and adolescence; and pre-registered protocols. The observation that an untreated control group improved as much as a treated group over five months [19] underlines that untreated or watchful-waiting comparators, rather than active comparators alone, are essential to determine which children, if any, receive clinically meaningful respiratory benefit from orthodontic interventions beyond medical management, and whether any benefit is durable over time.

## Conclusions

Selected orthodontic interventions may improve respiratory indices in appropriately diagnosed children with SDB, with comparatively stronger controlled evidence available for mandibular advancement appliances than for maxillary expansion. However, the available evidence remains heterogeneous and of low to very low certainty. Where maxillary expansion has been compared against an untreated concurrent control or against adenotonsillectomy, it has not shown a significant advantage. The evidence does not establish equivalence with established physician-directed therapies and does not support claims that orthodontic treatment prevents the future development of SDB.

Consistent with AAO and AAPD recommendations and, where applicable, informed by AADSM/AASM dental sleep medicine guidance and AAOP perspectives on interdisciplinary management, the orthodontist’s evidence-based role is to identify children at risk, conduct appropriate risk screening, and facilitate referral within an interdisciplinary, physician-led pathway. Physicians establish the diagnosis and direct medical management; polysomnography remains the diagnostic reference standard; and craniofacial imaging should not be used as a standalone screening or diagnostic test for pediatric SDB. The clinical framework presented in this systematic review translates these principles into practice while remaining aligned with the limitations of the current evidence.
